# Uncovering the complexity of childhood undernutrition through strain-level analysis of the gut microbiome

**DOI:** 10.1186/s12866-024-03211-w

**Published:** 2024-03-05

**Authors:** Bingmei Chang, Wenjie Zhang, Yinan Wang, Yuanzheng Zhang, Shilin Zhong, Peng Gao, Lili Wang, Zicheng Zhao

**Affiliations:** 1 Department of Biochemistry and Molecular Biology, Shanxi Medical University, Taiyuan, People’s Republic of China; 2 Department of Anesthesiology, First Hospital of Shanxi Medical University, Taiyuan, People’s Republic of China; 3 Peking University Shenzhen Hospital, Shenzhen, People’s Republic of China; 4 Shenzhen Byoryn Technology Co., Ltd, Shenzhen, People’s Republic of China; 5 BGI-Shenzhen, Beishan Industrial Zone, Shenzhen, People’s Republic of China

**Keywords:** Strain-level analysis, Childhood undernutrition, Gut microbiome, GM complex network, Nutrient absorption

## Abstract

**Background:**

Undernutrition (UN) is a critical public health issue that threatens the lives of children under five in developing countries. While evidence indicates the crucial role of the gut microbiome (GM) in UN pathogenesis, the strain-level inspection and bacterial co-occurrence network investigation in the GM of UN children are lacking.

**Results:**

This study examines the strain compositions of the GM in 61 undernutrition patients (UN group) and 36 healthy children (HC group) and explores the topological features of GM co-occurrence networks using a complex network strategy. The strain-level annotation reveals that the differentially enriched species between the UN and HC groups are due to discriminated strain compositions. For example, *Prevotella copri* is mainly composed of *P. copri* ASM1680343v1 and *P. copri* ASM345920v1 in the HC group, but it is composed of *P. copri* ASM346549v1 and *P. copri* ASM347465v1 in the UN group. In addition, the UN-risk model constructed at the strain level demonstrates higher accuracy (AUC = 0.810) than that at the species level (AUC = 0.743). With complex network analysis, we further discovered that the UN group had a more complex GM co-occurrence network, with more hub bacteria and a higher clustering coefficient but lower information transfer efficiencies. Moreover, the results at the strain level suggested the inaccurate and even false conclusions obtained from species level analysis.

**Conclusions:**

Overall, this study highlights the importance of examining the GM at the strain level and investigating bacterial co-occurrence networks to advance our knowledge of UN pathogenesis.

**Supplementary Information:**

The online version contains supplementary material available at 10.1186/s12866-024-03211-w.

## Background

Childhood undernutrition is a significant global public health issue contributing to approximately 45$\%$ of deaths in children under five years of age [1]. Undernutrition not only poses a high risk of mortality but also causes growth stunting, immune dysfunction, neurocognitive deficits, and endocrine system disorders [2, 3]. Although providing nutrient supplementation and antibiotics is the current therapeutic approach for undernutrition [4, 5], the long-term use of antibiotics is controversial, and alternative therapeutic strategies are necessary [6, 7].

In recent years, the gut microbiome (GM) has emerged as a promising target for the treatment of undernutrition due to its crucial role in nutrient metabolism and immune regulation. Previous studies have identified the association between GM composition and undernutrition. By studying a large child cohort in Bangladesh for two years, Subramanian et al. found the persistent immature GM in undernutrition (UN) children [7]; and the immature GM led to the mouse growth deficit after transferring to a mouse [6, 8]. Yan et al. reported that GM participated in the secretion of insulin-like growth factor 1 (IGF-1), promoting bone formation and growth [9]. Also, acetate, a short-chain fatty acid, released by the microbiome fermentation, affects the body’s adiposity [10]. However, these investigations have largely been conducted at the species or functional level, and the specific contributions of individual GM strains remain unclear.

To address this knowledge gap, we present a novel strategy to detect GM compositions at the strain level in undernourished children. Our approach leverages metagenomic sequencing and machine learning algorithms to identify specific GM strains associated with undernutrition. By examining the GM at the strain level, we aim to gain a more comprehensive understanding of the complex relationship between GM and undernutrition, potentially identifying new avenues for developing GM-based therapies for undernutrition.

## Methods

### Data preparation

The metagenomic sequencing data from 61 undernutrition patients (UN group) and 36 healthy children (HC group) was obtained from our previous study (the NCBI Sequence Read Archive Database under the accession number PRJNA543967) [11]. DNA libraries were sequenced with the 150 bp paired-end mode on the HiSeq platform (Illumina, San Diego, CA, United States). The concentrations of hemoglobin, albumin, total protein, white blood cells, and lymphocytes were measured by the automatic blood analyzer (Beckman Coulter AU5800, Brea, CA, USA) using peripheral blood. All recruited children were under three years old. The UN children had moderate or severe undernutrition, which was defined as having a weight-for-age z-score two standard deviations below the WHO reference value. The HC children were selected from those who passed physical examinations with no diarrhea in the past two weeks. Moreover, our previous study excluded participants who had been exposed to antibiotics, probiotics, or proton pump inhibitors the month before fecal sample collection, had a known history of allergies or hereditary diseases, suffered from metabolic or autoimmune diseases, or had parasitic eggs in their stool. The sequencing reads were filtered with the following criteria: 1) The raw reads containing more than 10 low-quality (<Q20) bases or 15 bases of adapter sequences were filtered out; 2) The raw reads which can be aligned to human genome hg19 by BWA-MEM were filtered out. After the filtration, the remaining reads were applied for the downstream analysis.

### Species taxonomic annotation

To obtain the taxonomic information for the samples, we applied the MetaPhlan3 software to align the clean data to the marker gene database (Version: mpa_v30_CHO- COPhlAn_201901) [12] and combine the taxonomical annotation files. We removed the species absent in 70$\%$ of the samples. At last, a total of 453 species remained for further analysis.

### Strain-level identification

We applied PStrain [13] to identify strains and infer strain abundances for each sample. Strains with a sequence similarity of more than 90$\%$ clustered together, and the identified strains were named by their cluster name. PStrain identified a total of 1,932 strain clusters. To annotate these strain clusters, we used PStrain-tracer [14] to obtain NCBI reference sequences for the species the strains belong to and construct the phylogenetic trees for the strain clusters and NCBI reference sequences [15]. Then, we used an in-house Python script to annotate the strain clusters with the closest NCBI known strains in the phylogenetic tree. With the strain profiling and identified strains, we completed the subsequential statistical analysis.

### Bacterial co-occurrence network and inner bacterial interactions

Based on the taxonomical profiling at the strain level, we calculated the Spearman correlation coefficient among strains by using the R software package “psych” [16]. The relations whose Spearman correlation coefficient <-0.6 or > 0.6 (adjusted P < 0.05) were kept as the edges for the co-occurrence network. In addition, we calculated the network topology information such as average degree, average path length, and cluster coefficient for each group. At last, the bacterial co-occurrence networks were visualized by Gephi (Version 0.9.2), with the nodes representing strains and edges representing the correlation between strains [17]. The species are colored by their corresponding phylum, and the red and pink edges stand for the positive and negative correlations, respectively. We further measured the network topological structure with the R package “igraph” on the constructed co-occurrence networks [18].

### Construction of Random Forest classifiers

Applying the Random Forest in machine learning (package “random-Forest” in R), we constructed classification models for the prediction of the UN and HC participants at the species and strain levels, respectively [19]. For the construction of the model, we randomly divided the total of 97 samples into five groups, with three groups containing 19 samples each and the other two groups containing 20 samples each. Then, we used the five-fold cross-validation method during the model construction. Four groups were selected as the training dataset, and one group was taken as the validation dataset randomly. Third, the Random Forest model was trained with the training dataset, and the performance of the constructed models was assessed with the validation dataset. At last, we plotted the Receiver Operating Characteristic (ROC) curves and calculated the AUC values to visualize the performance of the classifier (package “pROC” in R). In addition, the species and strains were recognized as candidate biomarkers for the UN prediction according to their Gini values and the optimal variation numbers in the Random Forest models.

### Statistical analysis

We evaluated the bacterial diversity using Shannon and Chao1 index at species and strain levels with R package “vegan” [20]. Then, we adopted the Wilcoxon rank sum test to explore the differentially enriched species and strains between different groups. The results were plotted with package “ggplot2” in R. In addition, we used Benjamini and Hochberg method (“p.adjust” in R) for the adjustment of the multiple Wilcoxon rank sum tests and Spearman correlation analysis (FDR<0.05) [21]. The Spearman correlation analysis results were visualized by the R package “pheatmap”. We denoted the nutrition-related clinical factors as nutrition indicators. Then we calculated the pairwise Pearson correlation between nutrition indicators, and the contribution of GM, short-chain fatty acids (SCFAs), and Weight to Age by the Mantel test [22]. The calculation and visualization were performed by the R package “linkET” [23].

## Results

### UN and HC children have similar strain diversity

**Fig. 1:**
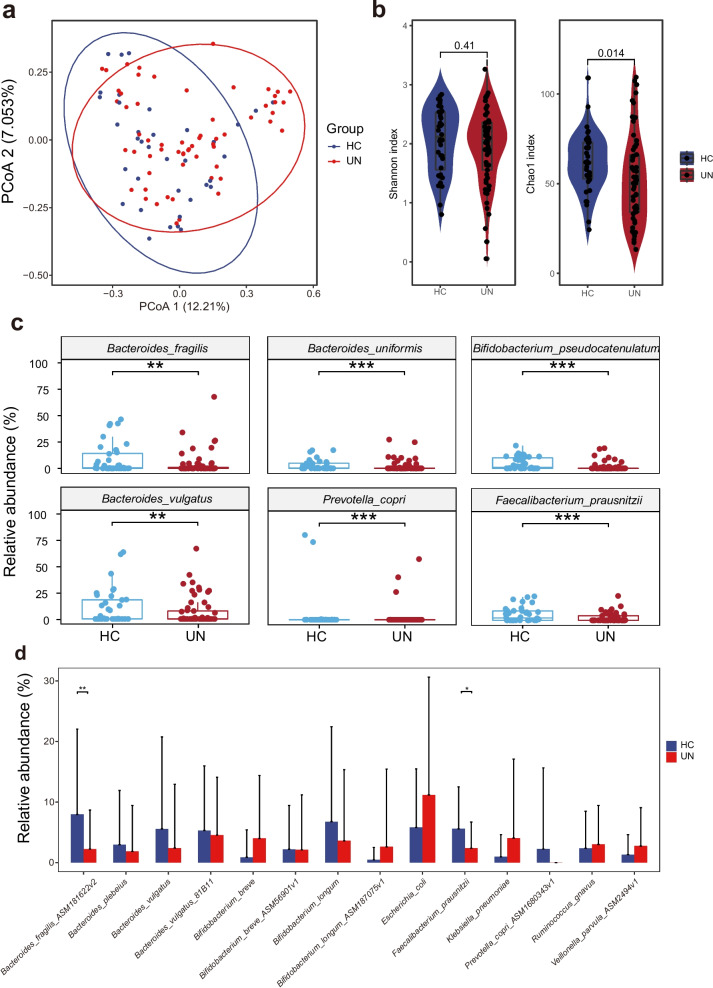
GM features in the UN patients. **a** PCoA with Bray-Curtis distances among samples. Each spot stands for a sample, and the circles contain samples with a 90$\%$ confidence interval for the groups. Red and blue colors stand for the UN and HC children, respectively. **b** Shannon diversity in the UN and HC children. The red and blue violins stand for the distributions of Shannon indices in the UN and HC groups, respectively. **c** Comparison of the top 5 abundant species between the UN and HC children. Each plot contained a species; the red and blue boxes stand for the UN and HC groups, respectively. **d** Comparison of the top 10 abundant strains between the UN and HC children. The red and blue columns stand for the UN and HC groups, respectively. *, Padj < 0.05; **, Padj < 0.01; ***, Padj < 0.001

To obtain the subtle bacterial signals related to the UN pathogenesis, we applied the PStrain software and detected the GM differences between the UN and HC children at the strain level [13]. The PCoA, Shannon and Chao1 diversity exhibited the overall GM differences between the UN and HC groups (Fig. 1a and b). The bacterial diversity in UN children was lower than that of HC children, although only Chao1 showed statistical differences (P = 0.01), while Shannon did not (P = 0.41, Fig. 1b). Then, we performed the abundance comparisons for the bacteria between the two groups at the species and strain levels, respectively. At the species level, we found six of the top ten abundant species were differentially enriched between UN and HC children (Fig. 1c). Among them, *Bacteroides fragilis* (Padj = 0.005), *Bacteroides uniformis* (Padj < 0.001), *Bifidobacterium pseudocatenulatum* (Padj < 0.001), *Faecalibacterium prausnitzii* (Padj < 0.001), and *Bacteroides vulgatus* (Padj = 0.008) were enriched in the HC group, while *Prevotella copri* (Padj < 0.001) was enriched in the UN group. Subsequently, we compared the differences of the top ten abundant strains between the UN and HC groups. We discovered that *B. fragilis* ASM181622v2 (Padj = 0.008) and *F. prausnitzii* (Padj = 0.012) were enriched in the HC group (Fig. 1d), indicating that the strain-level analysis would provide us with more comprehensive information for UN pathogenesis investigation.

### Strain compositional differences explain discriminated species in the UN children

**Fig. 2:**
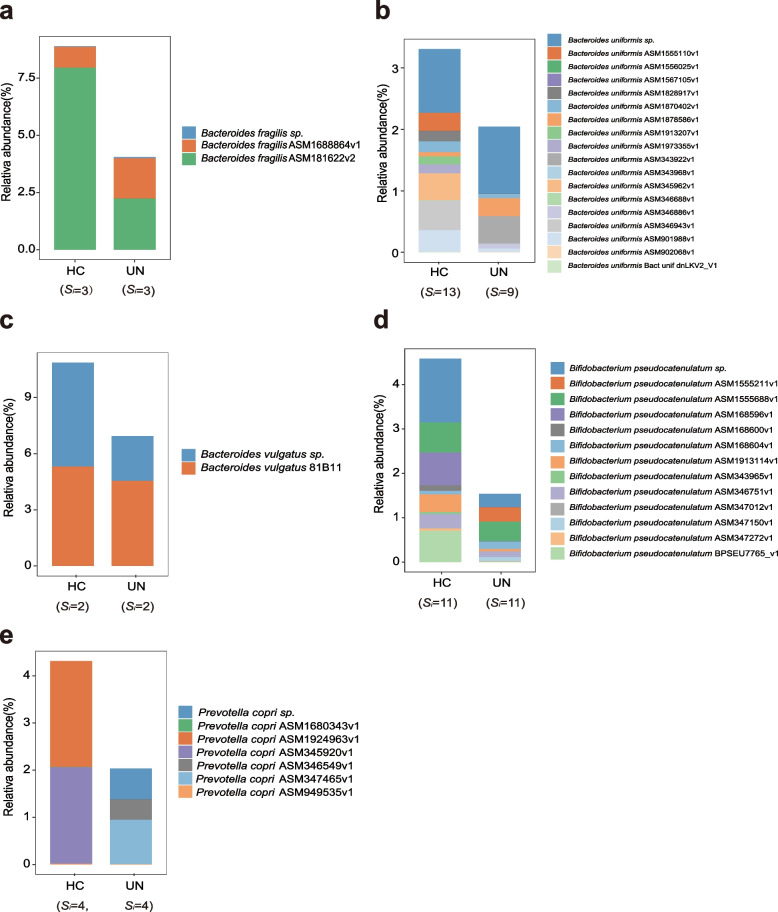
Strain components of the differentially enriched species in the UN patients. The plots **a**, **b**, **c**, **d**, and **e** exhibited the components and abundances of *Bacteroides fragilis*, *Bacteroides uniformis*, *Bacteroides vulgatus*, *Bifidobacterium pseudocatenulatum*, and *Prevotella copri* in the UN and HC groups, respectively. The number of the identified strains for each species was labeled below the plots for each group, and their abundances were represented by the height of the columns

With the NR database and PStrain-tracer software, we obtained the strain-level profiling and discovered the specific strain compositional characteristics for the differentially enriched species between the UN and HC children (Fig. 2, Supplementary Figs. 1, 2, 3, 4, and 5, and Supplementary Table 1). First, the strain composition of the same species in the UN patients was completely different from the HC children. For example, *P. copri* was mainly composed of *P. copri* ASM1680343v1 and *P. copri* ASM345920v1 in the HC group, while it was composed of *P. copri* ASM346549v1 and *P. copri* ASM347465v1 in the UN group (Fig. 2e). Second, the common strains between the UN and HC groups had different abundances, leading to species-level differences. For example, the enriched *B. fragilis* ASM181622v2 in the HC group was responsible for the *B. fragilis* discrimination between the UN and HC groups (Fig. 2a), while *B. vulgatus* sp. contributed to the *B. vulgatus* difference between the UN and HC groups (Fig. 2c). Meanwhile, highly abundant *B. pseudocatenulatum* BPSEU7765_v1, *B. pseudocatenulatum* ASM168596v1, and *B. pseudocatenulatum* ASM346751v1 in the HC group laid the basis for the enrichment of *B. pseudocatenulatum* in the HC group (Fig. 2d). Finally, the strain diversity for the same species might be different between the UN and HC groups. For example, the strain diversity of *B. uniformis* in the HC group (Si = 13) was higher than that in the UN group (Si=9, Fig. 2b). Based on these findings, we concluded that the strain-level annotation results can not only help us obtain subtle information on the GM compositions but also provide an important basis for further accurate GM intervention in diseases.

### The UN children exhibited a more complex but lower efficient GM co-occurrence network

**Fig. 3:**
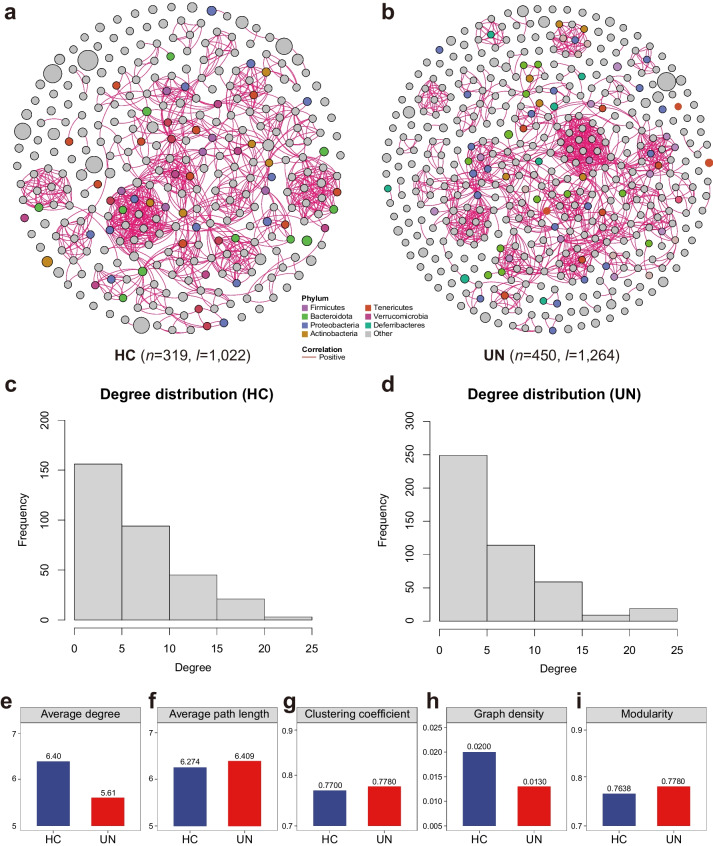
Topological features of the GM co-occurrence networks in the HC and UN patients. Plot **a** and **b** describe the GM co-occurrence networks in the HC and UN, respectively. The number of nodes and edges for the networks are labeled under each plot. Each circle stands for a strain, and its diameter represents the abundance of the strain. The lines stand for the correlations among the strains, while the positive correlations were marked with red color. Plot **c** and **d** describe the degree distributions of the nodes for the GM networks in the HC and UN groups. Plot **e**, **f**, **g**, **h**, and **i** exhibited the average degree, average path length, clustering coefficient, graph density, and modularity in the UN and HC groups, respectively

To further detect the hub bacteria that manipulate the GM, we constructed the GM co-occurrence networks for the UN and HC children and compared their topological features with a complex network approach. We discovered that the UN children had a larger network than the HC children, including more nodes and edges (Fig. 3a and b). In addition, the degree distributions of nodes in both UN and HC were confronted with the power-law distribution, implying that they are both scale-free networks (Fig. 3c and d). The GM network in HC children contained 19 hub nodes, including *Bacteroides stercoris* ASM343802v1, *Alistipes putredinis* ASM1913202v1, *Butyricicoccus pullicaecorum*, etc. (Fig. 3a, Supplementary Table 2). In UN children, the GM network contained 21 hub nodes, including *A. putredinis*, *Ruminococcus bromii* ASM346616v1, *Barnesiella intestinihominis*, etc. (Fig. 3b, Supplementary Table 3). The topological structure analysis indicated that the GM network in UN children has a higher clustering coefficient (0.7780 vs. 0.7700) and modularity (0.7780 vs. 0.7638), indicating their more complex network. However, the graph density (Fig. 3h) and the average degree of each bacterium (Fig. 3e) in the UN patients were lower than those in the HC children, and the average path length was longer in the UN children (Fig. 3f), suggesting the connectivity and information transferring efficiency was lower in UN patients than that of HC children. The finding suggested that the UN children had a discriminated GM network, providing us with bacterial candidates for further GM interventions in these patients.

### Improved predictive power of UN-risk model at the strain level

**Fig. 4:**
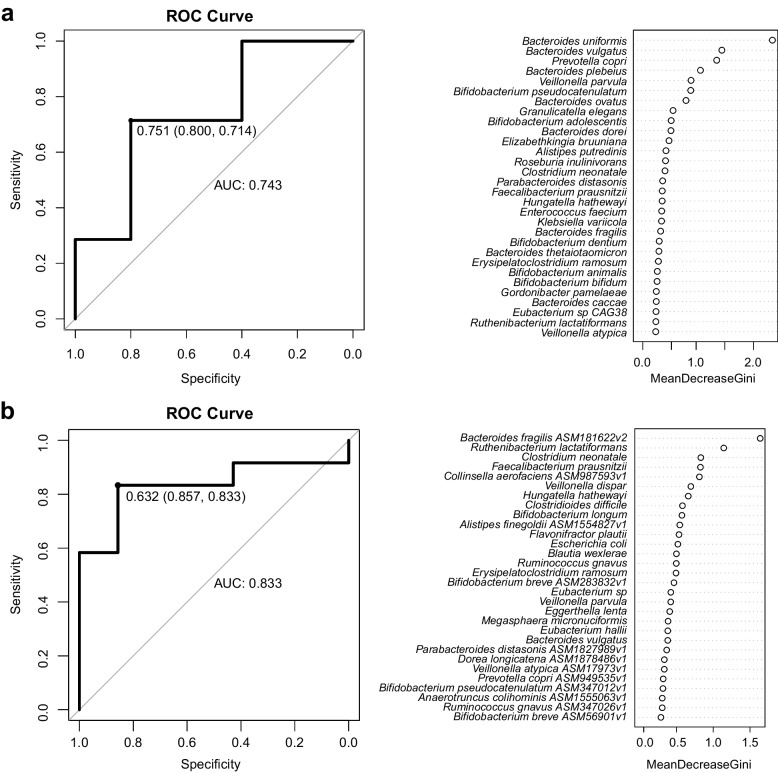
UN-risk models constructed with GM at the species and strain levels. **a** The ROC curves and Gini values for the UN-risk model constructed at the species level. *B. uniformis*, *B. vulgatus*, *P. copri*, *B. plebeius*, and *V. parvula* posed essential contributions to the construction of the model. **b** The ROC curves and Gini values for the UN-risk model constructed at the strain level. *B. fragilis* ASM181622v2, *R. lactatiformans*, *C. neonatale*, *F. prausnitzii*, and *C. aerofaciens* ASM987593v1 posed essential contributions to the con-struction of the model

With the random forest algorithm, we constructed the UN-risk models at species and strain levels, respectively, and identified microbial markers for the UN patients (Fig. 4). After constructing the UN-risk models, we recognized the top five variables with significant contributions as the potential biomarkers for the UN patient screening. At the species level, *B. uniformis*, *B. vulgatus*, and *P. copri* were the potential biomarkers (Fig. 4a). Whilst, *B. fragilis* ASM181622v2, *Ruthenibacterium lactatiformans*, and *Clostridium neonatale* sp. were the potential biomarkers at the strain level (Fig. 4b). The findings suggested that the potential biomarkers were uncoordinated at species and strain levels. In addition, the UN-risk model constructed from the strain compositions exhibited higher accuracy (area under curve, AUC = 0.833) than that from the specie compositions (AUC = 0.743), implying the higher accuracy UN-risk model constructed with strain profiling.

### Intimated correlations existed between GM, intestinal SCFAs, and nutritional indicators

**Fig. 5:**
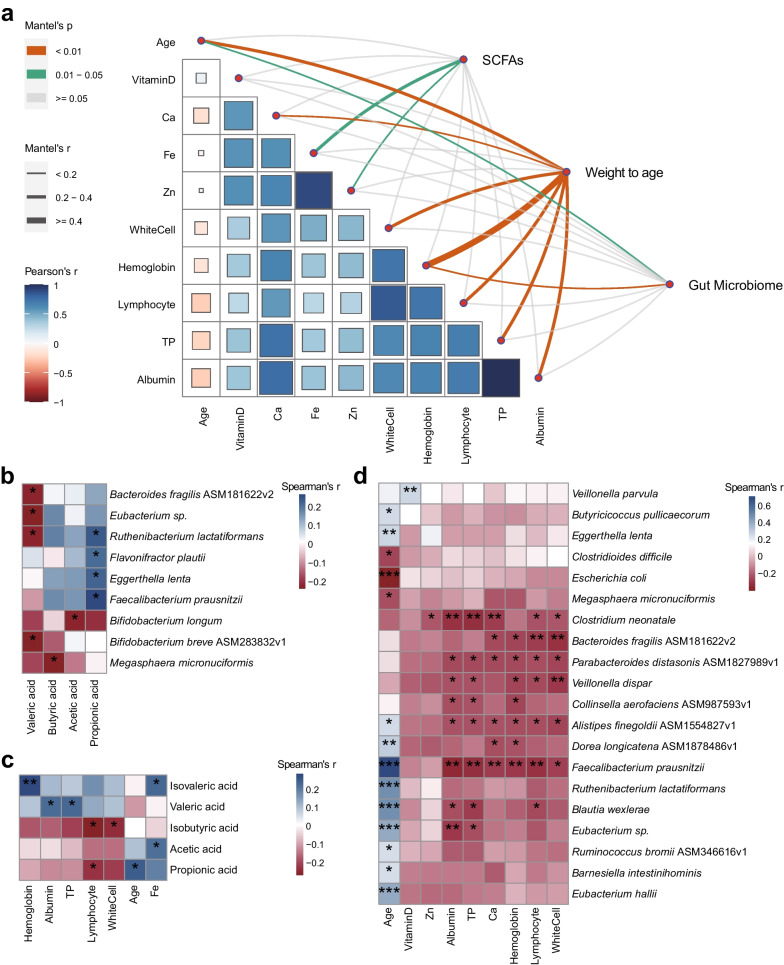
Correlations among the GM strains, fecal SCFAs, and the UN clinical indicators. **a** Contributions of SCFAs, Weight to age, and GM on the physiological indicators of UN. The thicker lines represent the higher contributions of variables on the physiological indicators. The oranges, green, and grey lines represent the adjusted *P*-value < 0.01, 0.01-0.05, and $\ge 0.05$, respectively. The blue and red boxes represent the positive and negative correlations among the clinical indicators, respectively. **b** Corre-lation heatmap between the SCFAs and GM strains. **c** Correlation heatmap between the SCFAs and the physiological indicators of UN. **d** Correlation heatmap between GM strains and the physiological indicators of UN. The blue and red boxes represent the positive and negative correlations, respectively. *, Padj < 0.05; **, Padj < 0.01; ***, Padj < 0.001

Based on Mentel analysis, we explored the general impacts of GM, weight to age, and short-chain fatty acids (SCFAs) on nutritional indicators in children, including leukocytes, lymphocytes, vitamin D, Ca, Fe, Zn, etc. (Fig. 5a). We first discovered that the GM was positively correlated with the age (Rpearson = 0.120, Padj = 0.027) and hemoglobin (Rpearson = 0.166, Padj = 0.001). Second, SCFAs were positively associated with the levels of Fe (Rpearson = 0.260, Padj = 0.014) and Zn (Rpearson = 0.155, Padj = 0.042). Third, weight to age, which is an important indicator for malnutrition clinical diagnosis, was positively correlated with other physical indicators, including the white cell number (Rpearson = 0.232, Padj = 0.001), the hemoglobin level (Rpearson = 0.502, Padj = 0.001), the lymphocyte number (Rpearson = 0.266, Padj = 0.001), the serum total protein (TP) level (Rpearson = 0.243, Padj = 0.001), the albumin level (Rpearson = 0.235, Padj = 0.001) and the Ca level (Rpearson = 0.186, Padj = 0.001, Fig. 5a). These findings implied the essential impact of GM and its derived SCFAs on nutrient absorption in humans and disclosed the intimate relationships between nutritional status and various physical indicators.

Then, we further investigated the detailed associations between GM, SCFAs, physical indicators, and trace elements in the children (Fig. 5b-d). We found that the levels of SCFAs were closely related to GM, e.g., propionic acid was positively correlated with *Ruthenibacterium lactatiformans* (Rspearman = 0.244, Padj = 0.024), *Flavonifractor plautii* (Rspearman = 0.222, Padj = 0.041), *Eggerthella lenta* (Rspearman = 0.230, Padj = 0.034), and *Faecalibacterium prausnitzii* (Rspearman = 0.273, Padj = 0.012, Fig. 5b). In addition, the associations between different SCFAs and the physical indicators varied in the children (Fig. 5c). Isovaleric acid was significantly positively correlated with the levels of hemoglobin (Rspearman = 0.291, Padj = 0.007) and Fe (Rspearman = 0.238, Padj = 0.028); Valeric acid was positively correlated with the levels of albumin (Rspearman = 0.230, Padj = 0.034) and TP (Rspearman = 0.237, Padj = 0.029); Isobutyric acid was negatively correlated with the numbers of lymphocyte (Rspearman = -0.261, Padj = 0.016) and white cell (Rspearman = -0.229, Padj = 0.035, Fig. 5c). Furthermore, GM is also closely related to physical indicators (Fig. 5d). Among them, *Veillonella parvula* was positively correlated with vitamin D (Rspearman = 0.230, Padj = 0.034); *Clostridium neonatale* was negatively correlated with the levels of Zn (Rspearman = -0.214, Padj = 0.050) and Ca (Rspearman = -0.280, Padj = 0.009); and *Faecalibacterium prausnitzii* was negatively correlated with albumin (Rspearman = -0.341, Padj = 0.001), TP (Rspearman = -0.324, Padj = 0.003), hemoglobin (Rspearman = -0.284, Padj = 0.010), lymphocyte (Rspearman = -0.305, Padj = 0.005), and white cell (Rspearman = -0.267, Padj = 0.014, Fig. 5d). The discoveries provided us with clinical references for the intervention of malnutrition through modulating GM or SCFAs modulations.

## Discussion

Numerous studies have investigated the gut microbiome in undernourished children to gain insight into the potential links between gut microbiota and undernutrition. However, these studies often overlook the strain-level diversity within bacterial species, which could provide additional information on the functional roles of specific bacterial strains in the pathogenesis of undernutrition [24, 25]. Our study addresses this gap in knowledge by analyzing the strain-level diversity of the gut microbiome in undernourished children and identifying specific strains that are differentially enriched between undernourished and healthy control children.

Our strain-level analysis revealed that undernourished children had enriched *Prevotella copri* strains, which have been linked to adverse conditions such as chronic inflammation [26], fat accumulation [27], insulin resistance [28], and glucose intolerance [28]. However, conflicting reports also associate *P. copri* with improved glucose and reduced visceral fat [29]. Our findings showed that the strains in undernourished and healthy children were from different clades, potentially having different metabolic roles. Higher taxonomic levels can obscure candidate bacteria that contribute to human diseases, and enriched pathogens at the strain level provide new clues to the pathogenesis of undernutrition.

We also highlighted the identified differences in GM composition between the UN and HC group, particularly the absence of certain beneficial bacterial strains in the UN group, highlighting potential associations between specific bacteria and undernutrition status. For instance, *Bacteroides fragilis* and *Bacteroides uniformis* strains are known to be involved in the fermentation of complex polysaccharides and the production of SCFAs [30–32]. Polysaccharide and oligosaccharide metabolism provides nutrition and vitamins to the host and other intestinal microbial residents [33]. SCFAs, such as butyrate, acetate, and propionate, serve as important energy sources for colonocytes, maintain intestinal homeostasis, and contribute significantly to host nutrition [33, 34]. Otherwise, *Faecalibacterium prausnitzii* is also one of the most important butyrate-producing bacteria [30]. The absence of *B. fragilis*, *B. uniformis*, and *F. prausnitzii *in undernourished children might result in inadequate production of SCFAs and reduced efficiency in polysaccharide metabolism. *B. fragilis ASM181622v2* might be one of the key strains. Our results also showed that *F. prausnitzii* was positively correlated with propionic and negatively correlated with albumin, TP, hemoglobin, lymphocyte, and white cells. This bacterium has potential roles in maintaining gut health. Moreover, the presence of lower levels of *Bifidobacterium pseudocatenulatum* strains, which are well-known for their beneficial effects on host health, in the UN group might suggest that undernourished children experienced impaired fermentation of dietary fibers, reduced production of short-chain fatty acids, and compromised immune function [30, 35–37]. These findings have significant implications for addressing the increasing rise of malnutrition. Understanding the specific roles of these bacteria in nutrient metabolism and gut health can potentially lead to interventions targeting the gut microbiome to alleviate undernutrition. Strategies such as dietary modifications (e.g., increasing dietary fiber and complex carbohydrates), probiotic supplementation with specific beneficial bacteria, or fecal microbiota transplantation may be explored to rebalance the gut microbiota and improve nutritional outcomes.

Co-occurrence network analysis revealed that different topological characteristics between UN and HC groups might be linked to different inter-species interactions or system efficiency [38]. Although the gut microbiota networks in both groups had scale-free properties, the hub nodes, which play a critical role in preserving the overall functionality of the network, were different [39–41]. Notably, the gut microbiota network of undernourished children exhibited higher clustering coefficient and modularity, along with lower graph density, lower average degree, and longer average path length. These characteristics are associated with lower community stability, worse microbial cooperation, and decreased communication [38, 40, 42].

Moreover, we constructed a UN risk model based on the strain compositions, demonstrating a better predictive effect than the model based on species compositions. At the species level, *Bacteroides vulgatus*, *Bacteroides uniformis*, and *Prevotella copri* were critical contributors to the risk prediction model and differentially enriched between undernourished and healthy children. However, at the strain level, their high intraspecific diversity resulted in no differentially enriched strains between the two groups. Critical strain biomarkers with significantly different abundances were *Bacteroides fragilis* ASM181622v2, *Ruthenibacterium lactatiformans*, and *Clostridium neonatale* sp., with critical biological functions in intestinal infection or homeostasis maintenance [43]. Our strain-level analysis provided a more precise UN risk model, enhancing our understanding of the associations between childhood undernutrition and GM.

## Conclusions

In conclusion, our study explored the characteristics of GM and its co-occurrence networks in undernourished children from the strain level perspective, supplementing the understanding of the GM’s roles in the pathogenesis of undernutrition, providing a more precise UN risk model, and laying the groundwork for undernutrition interventions.

### Supplementary Information

**Additional file 1.**

**Additional file 2.**

**Additional file 3.**

**Additional file 4.**

**Additional file 5.**

**Additional file 6.**

## Data Availability

The gut metagenomic data have been deposited in the NCBI Sequence Read Archive (SRA) repository under BioProject accession number PRJNA543967. All other data is available from the authors upon reasonable request.

## Abbreviations

UN: Undernutrition
GM: Gut microbiome
HC: Healthy
AUC: Area under curve
WHO: World Health Organization
IGF-1: Insulin-like growth factor 1
T2D: Type 2 diabetes
SNP: Single Nucleotide Polymorphism
SCFAs: Short chain fatty acids

## References

[1] Black RE, Victora CG, Walker SP, Bhutta ZA, Christian P, De Onis M (2013). Maternal and child undernutrition and overweight in low-income and middle-income countries. Lancet..

[2] de Onis M, Garza C, Victora CG, Onyango AW, Frongillo EA, Martines J (2004). The WHO Multicentre Growth Reference Study: planning, study design, and methodology. Food Nutr Bull..

[3] Prendergast AJ, Humphrey JH (2014). The stunting syndrome in developing countries. Paediatr Int Child Health..

[4] Trehan I, Goldbach HS, LaGrone LN, Meuli GJ, Wang RJ, Maleta KM (2016). Antibiotics as part of the management of severe acute malnutrition. Malawi Med J..

[5] Gough EK, Moodie EE, Prendergast AJ, Johnson SM, Humphrey JH, Stoltzfus RJ (2014). The impact of antibiotics on growth in children in low and middle income countries: systematic review and meta-analysis of randomised controlled trials. BMJ..

[6] Blanton LV, Charbonneau MR, Salih T, Barratt MJ, Venkatesh S, Ilkaveya O, et al. Gut bacteria that prevent growth impairments transmitted by microbiota from malnourished children. Science. 2016;351(6275). 10.1126/science.aad3311. 10.1126/science.aad3311 PMC4787260 26912898

[7] Subramanian S, Huq S, Yatsunenko T, Haque R, Mahfuz M, Alam MA (2014). Persistent gut microbiota immaturity in malnourished Bangladeshi children. Nature..

[8] Charbonneau MR, O’Donnell D, Blanton LV, Totten SM, Davis JC, Barratt MJ (2016). Sialylated Milk Oligosaccharides Promote Microbiota-Dependent Growth in Models of Infant Undernutrition. Cell..

[9] Yan J, Herzog JW, Tsang K, Brennan CA, Bower MA, Garrett WS (2016). Gut microbiota induce IGF-1 and promote bone formation and growth. Proc Natl Acad Sci U S A..

[10] Sahuri-Arisoylu M, Brody LP, Parkinson JR, Parkes H, Navaratnam N, Miller AD (2016). Reprogramming of hepatic fat accumulation and ‘browning’ of adipose tissue by the short-chain fatty acid acetate. Int J Obes (Lond)..

[11] Li D, Li Y, Dai W, Wang H, Qiu C, Feng S, et al. Intestinal Bacteroides sp. Imbalance Associated With the Occurrence of Childhood Undernutrition in China. Front Microbiol. 2019;10:2635. 10.3389/fmicb.2019.02635. 10.3389/fmicb.2019.02635 PMC6895006 31849851

[12] Truong DT, Franzosa EA, Tickle TL, Scholz M, Weingart G, Pasolli E (2015). MetaPhlAn2 for enhanced metagenomic taxonomic profiling. Nat Methods..

[13] Wang S, Jiang Y, Li S. PStrain: An Iterative Microbial Strains Profiling Algorithm for Shotgun Metagenomic Sequencing Data. Bioinformatics. 2020. 10.1093/bioinformatics/btaa1056. 10.1093/bioinformatics/btaa1056 33346799

[14] Jiang Y, Wang S, Wang Y, Zhang X, Li S. A framework to trace microbial engraftment at the strain level during fecal microbiota transplantation. bioRxiv. 2022. 10.1101/2022.05.18.492592.

[15] Schoch CL, Ciufo S, Domrachev M, Hotton CL, Kannan S, Khovanskaya R, et al. NCBI Taxonomy: a comprehensive update on curation, resources and tools. Database (Oxford). 2020. 10.1093/database/baaa062. 10.1093/database/baaa062 PMC7408187 32761142

[16] Revelle W. psych: Procedures for Psychological, Psychometric, and Personality Research. 2022. https://CRAN.R-project.org/package=psych.

[17] Bastian M, Heymann S, Jacomy M. Gephi: an open source software for exploring and manipulating networks 3(1), 361–362. Proceedings of the international AAAI conference on web and social media. 2009. 10.1609/icwsm.v3i1.13937.

[18] Nepusz GC. Tamas: The igraph software package for complex network research. Complex Syst. 2006. https://igraph.org.

[19] Wiener AL, Matthew. Classification and Regression by randomForest. R News. 2002. http://CRAN.R-project.org/doc/Rnews/.

[20] Oksanen J, Simpson GL, Blanchet FG, Kindt R, Legendre P, Minchin PR, et al. Vegan: Community Ecology Package. R package Version 2.6-4. 2022. https://cran.r-project.org/web/packages/vegan/index.html

[21] Grant GR, Liu J, Stoeckert JCJ (2005). A practical false discovery rate approach to identifying patterns of differential expression in microarray data. Bioinformatics..

[22] Borcard D, Legendre P (2012). Is the Mantel correlogram powerful enough to be useful in ecological analysis? A simulation study.. Ecology..

[23] Huang H. linkET: Everything is Linkable. R package version 0.0.3. 2021. https://github.com/Hy4m/linkET.

[24] Figler HM, Dudley EG (2016). The interplay of Escherichia coli O157:H7 and commensal E. coli: the importance of strain-level identification. Expert Rev Gastroenterol Hepatol..

[25] Nanjundiah V (2019). Many roads lead to Rome: Neutral phenotypes in microorganisms. J Exp Zool B Mol Dev Evol..

[26] Dillon SM, Lee EJ, Kotter CV, Austin GL, Gianella S, Siewe B (2016). Gut dendritic cell activation links an altered colonic microbiome to mucosal and systemic T-cell activation in untreated HIV-1 infection. Mucosal Immunol..

[27] Chen C, Fang S, Wei H, He M, Fu H, Xiong X (2021). Prevotella copri increases fat accumulation in pigs fed with formula diets. Microbiome..

[28] Pedersen HK, Gudmundsdottir V, Nielsen HB, Hyotylainen T, Nielsen T, Jensen BA (2016). Human gut microbes impact host serum metabolome and insulin sensitivity. Nature..

[29] Kovatcheva-Datchary P, Nilsson A, Akrami R, Lee YS, De Vadder F, Arora T (2015). Dietary Fiber-Induced Improvement in Glucose Metabolism Is Associated with Increased Abundance of Prevotella. Cell Metab..

[30] Rios-Covian D, Arboleya S, Hernandez-Barranco AM, Alvarez-Buylla JR, Ruas-Madiedo P, Gueimonde M (2013). Interactions between Bifidobacterium and Bacteroides species in cofermentations are affected by carbon sources, including exopolysaccharides produced by bifidobacteria. Appl Environ Microbiol..

[31] Rios-Covian D, Cuesta I, Alvarez-Buylla JR, Ruas-Madiedo P, Gueimonde M, de Los Reyes-Gavilán CG (2016). Bacteroides fragilis metabolises exopolysaccharides produced by bifidobacteria. BMC Microbiol..

[32] Rios-Covian D, Sánchez B, Salazar N, Martínez N, Redruello B, Gueimonde M (2015). Different metabolic features of Bacteroides fragilis growing in the presence of glucose and exopolysaccharides of bifidobacteria. Front Microbiol..

[33] Zafar H, Saier MH (2021). Gut Bacteroides species in health and disease. Gut Microbes..

[34] Wexler HM (2007). Bacteroides: the good, the bad, and the nitty-gritty. Clin Microbiol Rev..

[35] Chen J, Chen X, Ho CL (2021). Recent development of probiotic bifidobacteria for treating human diseases. Front Bioeng Biotechnol..

[36] O’Callaghan A, Van Sinderen D (2016). Bifidobacteria and their role as members of the human gut microbiota. Front Microbiol..

[37] Chung The H, Nguyen Ngoc Minh C, Tran Thi Hong C, Nguyen Thi Nguyen T, Pike LJ, Zellmer C, et al. Exploring the genomic diversity and antimicrobial susceptibility of Bifidobacterium pseudocatenulatum in a Vietnamese population. Microbiol Spectr. 2021;9(2):e00526–21. 10.1128/Spectrum.00526-21 PMC8557894 34523984

[38] Guo B, Zhang L, Sun H, Gao M, Yu N, Zhang Q, et al. Microbial co-occurrence network topological properties link with reactor parameters and reveal importance of low-abundance genera. npj Biofilms Microbiomes. 2022;8(1):3. 10.1038/s41522-021-00263-y PMC8764041 35039527

[39] Jing G, Zhang Y, Liu L, Wang Z, Sun Z, Knight R (2021). A scale-free, fully connected global transition network underlies known microbiome diversity. Msystems..

[40] Li Y, Chen Y, Fan Y, Chen Y, Chen Y (2023). Dynamic network modeling of gut microbiota during Alzheimer’s disease progression in mice. Gut Microbes..

[41] Vernocchi P, Gili T, Conte F, Del Chierico F, Conta G, Miccheli A (2020). Network analysis of gut microbiome and metabolome to discover microbiota-linked biomarkers in patients affected by non-small cell lung cancer. Int J Mol Sci..

[42] Loftus M, Hassouneh SAD, Yooseph S (2021). Bacterial associations in the healthy human gut microbiome across populations. Sci Rep..

[43] Uzal FA, Navarro MA, Li J, Freedman JC, Shrestha A, McClane BA (2018). Comparative pathogenesis of enteric clostridial infections in humans and animals. Anaerobe..

