# Correction: Uncovering the complexity of childhood undernutrition through strain‑level analysis of the gut microbiome

**DOI:** 10.1186/s12866-024-03287-4

**Published:** 2024-04-08

**Authors:** Bingmei Chang, Wenjie Zhang, Yinan Wang, Yuanzheng Zhang, Shilin Zhong, Peng Gao, Lili Wang, Zicheng Zhao

**Affiliations:** 1 Department of Biochemistry and Molecular Biology, Shanxi Medical University, Taiyuan, People’s Republic of China; 2 Department of Anesthesiology, First Hospital of Shanxi Medical University, Taiyuan, People’s Republic of China; 3 Peking University Shenzhen Hospital, Shenzhen, People’s Republic of China; 4 Shenzhen Byoryn Technology Co., Ltd, Shenzhen, People’s Republic of China; 5 BGI‑Shenzhen, Beishan Industrial Zone, Shenzhen, People’s Republic of China

**Correction: ***BMC Microbiol ***24**, **73 (2024)**

10.1186/s12866-024-03211-w

Following publication of the original article [1], the authors identified an error in the order of author names in author list and its affiliations. The incorrect and correct author list, as well as its affiliation, are provided below. The original article has been corrected.

The incorrect author list:

Lili Wang^1^, Wenjie Zhang^1^, Yinan Wang^2^, Yuanzheng Zhang^3^, Shilin Zhong^2^, Peng Gao^4*^, Bingmei Chang^5*^ and Zicheng Zhao^3*^.

The incorrect affiliations:

1 Department of Anesthesiology, First Hospital of Shanxi Medical University, Taiyuan, People’s Republic of China.

2 Peking University Shenzhen Hospital, Shenzhen, People’s Republic of China.

3 Shenzhen Byoryn Technology Co., Ltd, Shenzhen, People’s Republic of China.

4 BGI Shenzhen, Beishan Industrial Zone, Shenzhen, People’s Republic of China.

5 Department of Biochemistry and Molecular Biology, Shanxi Medical University, Taiyuan, People’s Republic of China.

The correct author list:

Bingmei Chang^1^, Wenjie Zhang^2^, Yinan Wang^3^, Yuanzheng Zhang^4^, Shilin Zhong^3^, Peng Gao^5*^, Lili Wang^2*^, and Zicheng Zhao^4*^.

The correct affiliations:

1 Department of Biochemistry and Molecular Biology, Shanxi Medical University, Taiyuan People’s Republic of China.

2 Department of Anesthesiology First Hospital of, Shanxi Medical University, Taiyuan People’s Republic of China.

3 Peking University Shenzhen Hospital, Shenzhen, People’s Republic of China.

4 Shenzhen Byoryn Technology Co., Ltd, Shenzhen, People’s Republic of China.

5 BGI-Shenzhen, Beishan Industrial Zone, Shenzhen, People’s Republic of China.

## References

[1] Wang L, Zhang W, Wang Y (2024). Uncovering the complexity of childhood undernutrition through strain-level analysis of the gut microbiome. BMC Microbiol.

