# Supplementary material for: Uncovering the complexity of childhood undernutrition through strain-level analysis of the gut microbiome
Source: BMC Microbiol. 2024 Mar 5;24:73. doi: 10.1186/s12866-024-03211-w (PMC10916198; doi:10.1186/s12866-024-03211-w)
Supplement: Supplementary file 2 — Additional file 2. [file 12866_2024_3211_MOESM2_ESM.pdf]

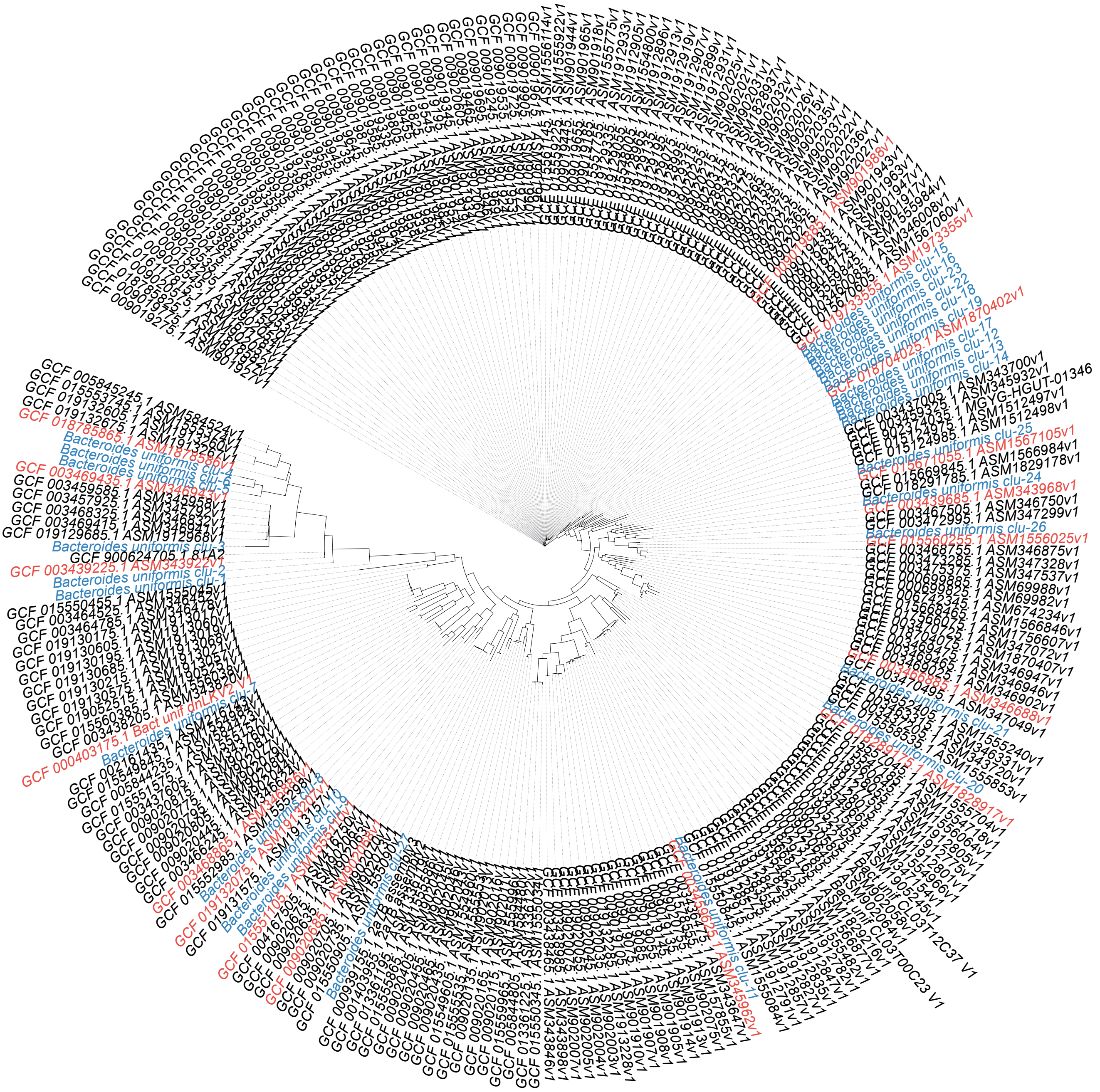

Bacteroides uniformis

Supplementary Figure 2. The phylogenetic relations of the strains in *Bacteroides uniformis*. The blue and red labeled strains represent the PStrain-identified strain clusters and their annotated strains in the NCBI, respectively. The black labeled strains represent other strains of the *Bacteroides uniformis* in the NCBI database.
