# Supplementary material for: Uncovering the complexity of childhood undernutrition through strain-level analysis of the gut microbiome
Source: BMC Microbiol. 2024 Mar 5;24:73. doi: 10.1186/s12866-024-03211-w (PMC10916198; doi:10.1186/s12866-024-03211-w)
Supplement: Supplementary file 5 — Additional file 5. [file 12866_2024_3211_MOESM5_ESM.pdf]

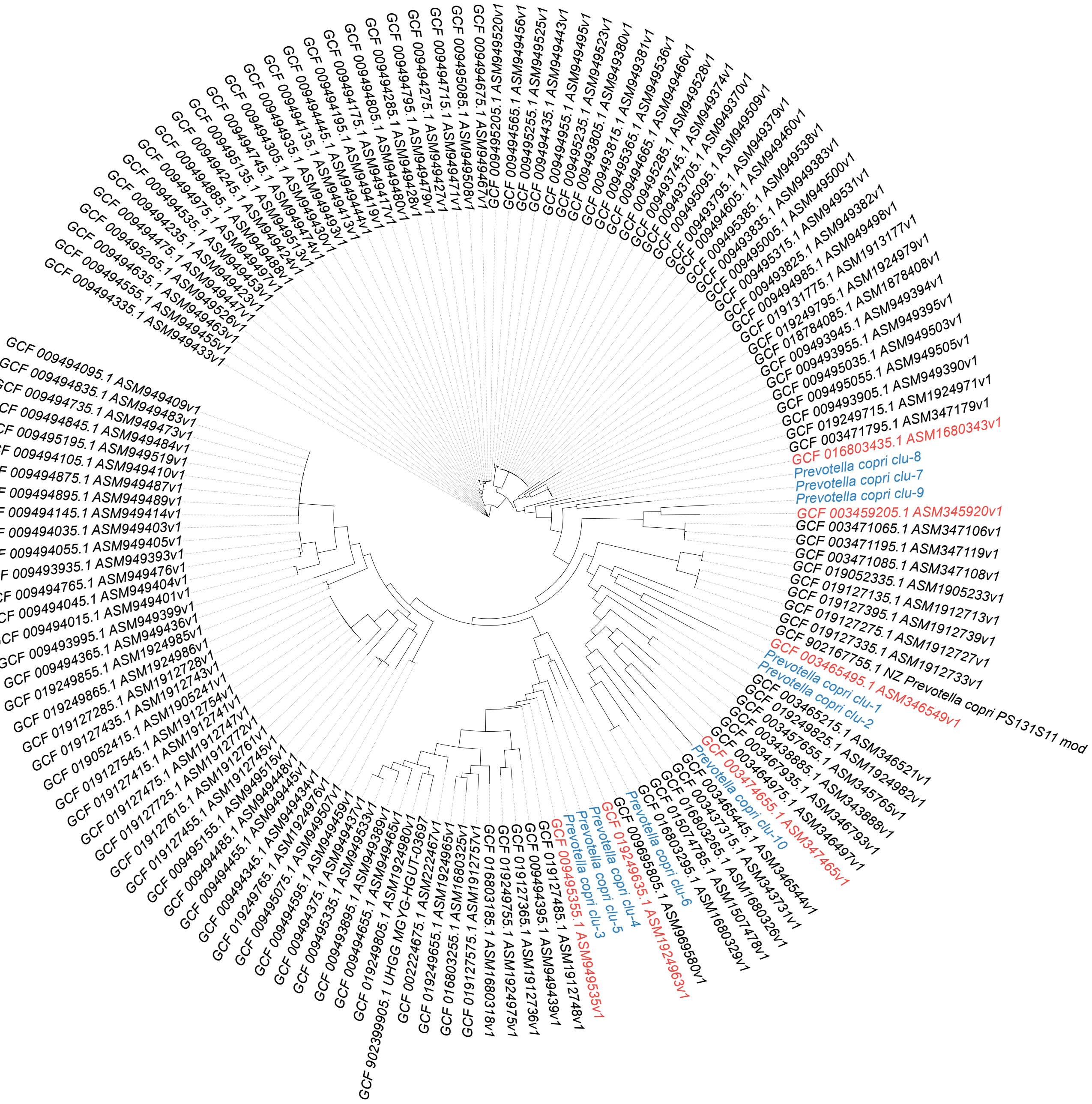

Prevotella copri

Supplementary Figure 5. The phylogenetic relations of the strains in *Prevotella copri*. The blue and red labeled strains represent the PStrain-identified strain clusters and their annotated strains in the NCBI, respectively. The black labeled strains represent other strains of the *Prevotella copri* in the N-CBI database.
